# COVIDiSTRESS Global Survey dataset on psychological and behavioural consequences of the COVID-19 outbreak

**DOI:** 10.1038/s41597-020-00784-9

**Published:** 2021-01-04

**Authors:** Yuki Yamada, Dominik-Borna Ćepulić, Tao Coll-Martín, Stéphane Debove, Guillaume Gautreau, Hyemin Han, Jesper Rasmussen, Thao P. Tran, Giovanni A. Travaglino, COVIDiSTRESS Global Survey Consortium, Angélique M. Blackburn, Loïs Boullu, Mila Bujić, Grace Byrne, Marjolein C. J. Caniëls, Ivan Flis, Marta Kowal, Nikolay R. Rachev, Vicenta Reynoso-Alcántara, Oulmann Zerhouni, Oli Ahmed, Rizwana Amin, Sibele Aquino, João Carlos Areias, John Jamir Benzon R. Aruta, Dastan Bamwesigye, Jozef Bavolar, Andrew R. Bender, Pratik Bhandari, Tuba Bircan, Huseyin Cakal, Tereza Capelos, Jiří Čeněk, Brendan Ch’ng, Fang-Yu Chen, Stavroula Chrona, Carlos C. Contreras-Ibáñez, Pablo Sebastián Correa, Irene Cristofori, Wilson Cyrus-Lai, Guillermo Delgado-Garcia, Eliane Deschrijver, Carlos Díaz, İlknur Dilekler, Vilius Dranseika, Dmitrii Dubrov, Kristina Eichel, Eda Ermagan-Caglar, Rebekah Gelpí, Rubén Flores González, Amanda Griffin, Moh Abdul Hakim, Krzysztof Hanusz, Yuen Wan Ho, Dayana Hristova, Barbora Hubena, Keiko Ihaya, Gozde Ikizer, Md. Nurul Islam, Alma Jeftic, Shruti Jha, Fernanda Pérez-Gay Juárez, Pavol Kacmar, Kalina Kalinova, Phillip S. Kavanagh, Mehmet Kosa, Karolina Koszałkowska, Raisa Kumaga, David Lacko, Yookyung Lee, Antonio G. Lentoor, Gabriel A. De Leon, Shiang-Yi Lin, Samuel Lins, Claudio Rafael Castro López, Agnieszka E. Lys, Samkelisiwe Mahlungulu, Tsvetelina Makaveeva, Salomé Mamede, Silvia Mari, Tiago A. Marot, Liz Martinez, Dar Meshi, Débora Jeanette Mola, Sara Morales-Izquierdo, Arian Musliu, Priyanka A. Naidu, Arooj Najmussaqib, Jean C. Natividade, Steve Nebel, Jana Nezkusilova, Irina Nikolova, Manuel Ninaus, Valdas Noreika, María Victoria Ortiz, Daphna Hausman Ozery, Daniel Pankowski, Tiziana Pennato, Martin Pírko, Lotte Pummerer, Cecilia Reyna, Eugenia Romano, Hafize Sahin, Aybegum Memisoglu Sanli, Gülden Sayılan, Alessia Scarpaci, Cristina Sechi, Maor Shani, Aya Shata, Pilleriin Sikka, Nidhi Sinha, Sabrina Stöckli, Anna Studzinska, Emilija Sungailaite, Zea Szebeni, Benjamin Tag, Mihaela Taranu, Franco Tisocco, Jarno Tuominen, Fidan Turk, Muhammad Kamal Uddin, Ena Uzelac, Sara Vestergren, Roosevelt Vilar, Austin Horng-En Wang, J. Noël West, Charles K. S. Wu, Teodora Yaneva, Yao-Yuan Yeh, Andreas Lieberoth

**Affiliations:** 1 Kyushu University, Fukuoka, Japan; 2 Catholic University of Croatia, Zagreb, Croatia; 3 University of Granada, Granada, Spain; 4 Independent researcher, La Mure, France; 5 Université Paris-Saclay, Juvisy sur Orge, France; 6 University of Alabama, Tuscaloosa, Alabama United States; 7 Aarhus University, Aarhus, Denmark; 8 Colorado State University, Fort Collins, Colorado United States; 9 University of Kent, Canterbury, UK; 10 Texas A&M International University, Laredo, Texas USA; 11 Tampere University, Tampere, Finland; 12 Vrije Universiteit Amsterdam, Amsterdam, the Netherlands; 13 Open University, Heerlen, The Netherlands; 14 University of Wrocłąw, Wrocław, Poland; 15 Sofia University StKliment Ohridski, Sofia, Bulgaria; 16 University of Veracruz; National Autonomous University of Mexico, Veracruz, Mexico; 17 Université Paris Nanterre, Nanterre, France; 18 University of Chittagong, Chittagong, Bangladesh; 19 Bahria University, Islamabad, Pakistan; 20 Pontifical Catholic University of Rio de Janeiro, Rio de Janeiro, Brazil; 21 University of Porto, Porto, Portugal; 22 De La Salle University, Manila, Philippines; 23 Mendel University in Brno, Brno, Czech Republic; 24 Pavol Jozef Safarik University, Košice, Slovakia; 25 Michigan State University, East Lansing, Michigan USA; 26 Saarland University, Saarbrücken, Germany; 27 Vrije Universiteit Brussel, Brussels, Belgium; 28 Keele University, Keele, UK; 29 University of Birmingham, Birmingham, UK; 30 University of Malaya, Kuala Lumpur, Malaysia; 31 King’s College London, London, United Kingdom; 32 Universidad Autónoma Metropolitana, Mexico City, Mexico; 33 Instituto de Investigaciones Psicológicas (IIPsi), Universidad Nacional de Córdoba (UNC), Consejo Nacional de Investigaciones Científicas y Técnicas (CONICET), Córdoba, Argentina; 34 University Claude Bernard Lyon 1; Institute of Cognitive Sciences Marc Jeannerod, CNRS UMR5229 Bron, France; 35 INSEAD, 1 Ayer Rajah Avenue, Singapore; 36 Instituto Nacional de Neurologia y Neurocirugia, Mexico City, Mexico; 37 Ghent University; University of New South Wales (UNSW), Belgium, Australia; 38 TOBB University of Economics and Technology, Ankara, Turkey; 39 Kaunas University of Technology, Kaunas, Lithuania; 40 National Research University Higher School of Economics, Moscow, Russian Federation; 41 Brown University, Providence, Rhode Island United States of America; 42 University of Northampton, Northampton, UK; 43 University of Toronto, Toronto, Canada; 44 University of Veracruz, Veracruz, Mexico; 45 Univeristy of Oregon, Eugene, Oregon, USA; 46 Universitas Sebelas Maret, Surakarta, Central Java, Indonesia; 47 Institute of Psychology Polish Academy of Sciences, Warsaw, Poland; 48 Northeastern University, Boston, Massachusetts, USA; 49 University of Vienna, Vienna, Austria; 50 International Christian University, Tokyo, Japan; 51 Somerville School (Lott Carey Baptist Mission in India), Noida, India; 52 McGill University, McGill, Canada; 53 University of Canberra, Canberra, Australia; 54 Tilburg University, Tilburg, Netherlands; 55 University of Lodz, Łódź, Poland; 56 University of Essex, Colchester, UK; 57 Masaryk university, Brno, Czech Republic; 58 The University of Texas at Austin, Austin, Texas USA; 59 Sefako Makgatho Health Sciences University, Pretoria North, Gauteng Province, South Africa; 60 The Education University of Hong Kong, Hong Kong, SAR Hong Kong; 61 University of Warsaw, Warsaw, Poland; 62 University of Milano-Bicocca, Milan, Italy; 63 University of California, Merced, USA; 64 University of Warwick, Warwick, United Kingdom; 65 Ludwig Maximilian University, Munich, Germany; 66 Griffith University, Brisbane, Australia; 67 Technische Universität Chemnitz, Chemnitz, Germany; 68 Leibniz-Institut für Wissensmedien, Tubingen, Germany; 69 University of Cambridge, Cambridge, UK; 70 California State University, Northridge, USA; 71 University of Economics and Human Sciences in Warsaw, Warsaw, Poland; 72 School of Compared Psychotherapy, Florence, Italy; 73 Institute of Lifelong Learning at Mendel University in Brno, Brno, Czech Republic; 74 Middle East Technical University, Ankara, Turkey; 75 Ankara Yıldırım Beyazıt University, Ankara, Turkey; 76 University of Cagliari, Cagliari, Sardinia Italy; 77 Hebrew University of Jerusalem, Jerusalem, Israel; 78 University of Miami, Coral Gables, Florida USA; 79 University of Skövde, Skövde, Sweden; 80 Indian Institute of Technology, Hyderabad, India; 81 University of Bern, Bern, Switzerland; 82 University of Helsinki, Helsinki, Finland; 83 University of Melbourne, Melbourne, Australia; 84 Universidad de Buenos Aires, Buenos Aires, Argentina; 85 University of Turku, Turku, Finland; 86 University of Sheffield, Sheffield, UK; 87 University of Dhaka, Dhaka, Bangladesh; 88 Faculty of Humanities and Social Sciences in Zagreb, Zagreb, Croatia; 89 University of Salford, Salford, UK; 90 Faculdades Integradas de Patos, Campina Grande, Brazil; 91 University of Nevada, Nevada, Las Vegas USA; 92 Purdue University, West Lafayette, Indiana, USA; 93 University of StThomas, Saint Paul, Minnesota USA

**Keywords:** Psychology, Human behaviour

## Abstract

This N = 173,426 social science dataset was collected through the collaborative COVIDiSTRESS Global Survey – an open science effort to improve understanding of the human experiences of the 2020 COVID-19 pandemic between 30th March and 30th May, 2020. The dataset allows a cross-cultural study of psychological and behavioural responses to the Coronavirus pandemic and associated government measures like cancellation of public functions and stay at home orders implemented in many countries. The dataset contains demographic background variables as well as measures of Asian Disease Problem, perceived stress (PSS-10), availability of social provisions (SPS-10), trust in various authorities, trust in governmental measures to contain the virus (OECD trust), personality traits (BFF-15), information behaviours, agreement with the level of government intervention, and compliance with preventive measures, along with a rich pool of exploratory variables and written experiences. A global consortium from 39 countries and regions worked together to build and translate a survey with variables of shared interests, and recruited participants in 47 languages and dialects. Raw plus cleaned data and dynamic visualizations are available.

## Abstract (summary)

**Table:** 

Measurement(s)	psychological measurement • anxiety-related behavior trait • Stress • response to • Isolation • loneliness measurement • Emotional Distress
Technology Type(s)	Survey
Factor Type(s)	geographic location • language • age of participant • responses to the Coronavirus pandemic
Sample Characteristic - Organism	Homo sapiens
Sample Characteristic - Location	global

Machine-accessible metadata file describing the reported data: 10.6084/m9.figshare.13251776

## Background & Summary

In 2020, a new coronavirus pandemic spread across countries worldwide. This resulted not only in a global health crisis, but also in severe economic and socio-psychological consequences. To control the spread of the coronavirus, governments imposed a range of measures, including the closure of schools, workplaces, shopping areas and public amenities, forced isolation, virus-testing, and limits to civil liberties. Inevitably, these changes generated a variety of psychological responses in individuals, which in turn shaped the level of compliance with preventive measures. In fact, extant research on the factors that shape willingness to comply with public health efforts aimed at preventing or slowing the spread of epidemics has highlighted the importance of psychological and social factors^1,2^—for instance shared trust in state or health authorities^3,4^—in driving compliance with guidelines and restrictions. The implications of these complex factors to compliance with preventive measures imposed by different governments must be analysed in detail after the crisis. Indeed, the psychological and societal effects are likely to be more pronounced, more widespread, and longer-lasting than the purely somatic effects of the infection^5^.

To contribute to the understanding of the intersection between pandemic-related physical and behavioural issues, the present document describes a large-scale dataset collected through the collaborative COVIDiSTRESS global survey. The COVIDiSTRESS data collection efforts ran from 30th March to 30th May, 2020 by collaborators from 39 countries and regions with survey forms available in 47 languages and dialects. In total, 173,426 participants were recruited from 179 countries on six continents.

Pandemic outbreaks breed misinformation, and foster fear of contagion as well as uncertainty during the course of their spread^5,6^. Factors such as concerns regarding the severity of a disease, the perceived reliability of government information, and beliefs in the efficacy of preventive measures can influence individuals’ intentions to comply and engage in preventive behaviours^7^. Thus, the extent of compliance is influenced by the level of trust in one’s sources of information about a pandemic, as well as the perceived gravity of the disease. Concerns over one’s risk of contracting the disease during a pandemic can be a source of ongoing worry and anxiety as well as stress (e.g. H1N1^7^ and MERS^8^). These concerns, as well as the confusion generated by the lack of established worldwide or national quarantine protocols, timely information and resources from public health systems^9^ may contribute to lower levels of compliance. Research indicates that the perception of openness and reliability of governments and health organisations^10^, levels of trust in media and medical authorities^11,12^ as well as perceptions of disease’s severity and the efficacy of one’s actions^10,13,14^ contribute to compliance with recommendations for preventive behaviour.

Both the medical situation and the psychological effects of isolation, confinement and information behavior^15,16^ need to be considered when prolonged periods of quarantine are implemented. A subset of negative effects on ‘cabin fever’ includes responses varying from anxiety and depression^17^ to impaired cognitive ability and hostility^16,18^. Efforts such as closing down schools and workplaces, and calls for people to self-isolate in their homes, are likely to constitute a source of both existential and practical stress unrelated to the fear of contracting the disease. Compliance with medical guidelines has been shown to decrease not just as a result of higher stress levels^19^, but also of minor everyday stressors such as workplace conflict or household responsibilities^20^. Prolonged states of emergency and the chronic psychological, social, and economic stressors related to them^21,22^ may decrease compliance with set behavioural objectives during pandemics. Conversely, social support from groups such as one’s family, friends, and colleagues moderate the effect of concern for the disease or other sources of stress on one’s psychological well-being^23,24^.

Hence, as an effort to help health authorities and decision makers organize informed responses, we initiated the COVIDiSTRESS open science collaboration. The dataset can help researchers and stakeholders identify nuances in psychological and behavioural risk factors in the context of the COVID-19 pandemic, and assist governments and other organizations in adopting constructive policies appropriate to each country.

## Methods

### Participants

173,426 people accessed an online survey link to provide their experiences over a period of 62 days (30th March to 30th May. The stored dataset represents 125,306 people who met inclusion criteria (18 years of age and older and gave informed consent). Demographic characteristics for countries with over 200 responses appear in Table 1. Given the urgent call for COVID-19 research, the survey received a waiver to commence data collection from the IRB office at Aarhus University, Denmark. Participants volunteered based on online and media appeals without monetary compensation; excepting some of the Japanese participants received 7 T-points (equivalent to about 0.065 USD) from the crowdsourcing service as a reward.

**Table 1:** Sample size, proportions of valid data, age mean and standard deviation across countries with more than 200 participants.

Country	N	Prop_50	Prop_90	M_age	SD_age
Finland	22933	0.854	0.804	43.357	14.170
France	13475	0.833	0.778	33.267	12.760
Denmark	10891	0.817	0.754	42.543	14.277
Mexico	9169	0.791	0.722	37.453	13.830
Lithuania	8255	0.796	0.720	38.553	12.459
Argentina	5923	0.711	0.598	41.593	15.244
Japan	5072	0.910	0.875	44.369	11.312
Bulgaria	4785	0.780	0.675	41.636	13.510
Poland	3088	0.779	0.694	31.315	7.883
Sweden	3055	0.825	0.764	46.477	12.373
Croatia	2965	0.807	0.739	35.408	12.247
Taiwan	2745	0.830	0.752	33.072	11.332
Kosovo	2707	0.615	0.468	29.225	10.058
United States	2314	0.832	0.783	42.857	14.714
Czech Republic	1995	0.787	0.720	33.375	11.506
Italy	1749	0.805	0.723	44.747	15.311
Indonesia	1569	0.723	0.616	31.047	9.572
United Kingdom	1500	0.775	0.701	39.438	12.814
Germany	1443	0.814	0.758	36.711	12.055
Hungary	1438	0.743	0.654	49.022	15.133
Netherlands	1433	0.800	0.748	44.944	14.730
Bosnia and Herzegovina	1288	0.780	0.661	37.256	11.972
Turkey	1199	0.760	0.667	33.533	11.809
Switzerland	1188	0.810	0.757	42.698	17.172
Portugal	1067	0.712	0.630	33.767	13.598
Slovakia	942	0.741	0.667	41.879	12.903
Panama	759	0.735	0.632	39.486	14.635
Brazil	731	0.778	0.703	35.259	13.748
Greece	642	0.822	0.745	41.785	11.622
Belgium	622	0.826	0.756	36.466	12.827
Spain	615	0.761	0.676	38.787	15.405
Philippines	570	0.849	0.777	25.853	11.424
Malaysia	567	0.769	0.709	36.795	14.411
Korea, South	487	0.764	0.671	38.053	10.427
Canada	470	0.811	0.760	41.349	14.540
Bangladesh	421	0.675	0.523	28.088	6.230
Pakistan	360	0.631	0.511	27.053	8.728
Australia	327	0.807	0.749	42.648	13.963
Austria	319	0.743	0.661	38.473	11.717
Romania	282	0.699	0.638	34.053	9.479
Serbia	266	0.816	0.688	38.556	12.651
Ireland	216	0.769	0.667	40.565	10.536

Note.

N = number of participants; Prop = proportion.

Prop_50 = proportion of participants that have more than 50% of non-missing data.

Prop_90 = proportion of participants that have more than 90% of non-missing data.

M_age = mean age; SD_age = standard deviation of age.

### Materials

The full survey form in English can be accessed at 10.17605/OSF.IO/Z39US. The survey consisted of two parts. The first section comprised general demographic data, self-reports about the proximate effects of the COVID-19 pandemic (e.g. isolation status, first-hand experience, attenuated risk), modified version of the Asian Disease problem to examine participants’ risk taking intention under COVID-19 situation^25^, personality assessment (BFI-S^26^), Short self-report scale of loneliness^27^ (SLON-3) based on the UCLA loneliness scale, Perceived Stress Scale (PSS-10^28^), self-reports about the interpersonal and institutional trust (based on OECD guidelines 2017), and items measuring daily behaviours including compliance with general and social preventive measures. The second part contained sets of more specific items related to people’s experiences of distress and worry during the ongoing outbreak of coronavirus (e.g. access to amenities, loss of work, adapting work, education and social interactions to digital platforms, the social stresses of confinement with adults and children), as well as items which detected copying mechanisms of people during the COVID-19 crisis (e.g. social contact, staying informed, dedicating oneself to preparation, hobbies, religion) and the Social Provisions Scale (SPS-10^29^). Finally, participants were asked to report information behaviours in times of the coronavirus pandemic, and were invited to add a few lines of text, to illuminate their experience of the COVID-19 crisis beyond the closed-end items. Participants typically supplied their answers on a 6-point Likert scale ranging from ‘Strongly disagree’ to ‘Strongly agree’, with some variation based on established standards, as well as in text boxes to add other relevant factors. Validated short versions of established measures were used if available in local languages. The full list of variables included in the COVIDiSTRESS global survey as well as the response options participants used to answer the survey are available at https://osf.io/v68t9/. To protect participants’ data and avoid sensitive information, participants were not asked about COVID-19 symptoms or other aspects of their medical status. Additionally, no data that would allow identification of participants was collected.

### Translation

The survey was translated into 47 languages and adapted to the dialects and vernacular of different regions (Afrikaans, Albanian, Arabic, Bangla, Indonesian, Bosnian, Bulgarian, Chinese [Simplified and Traditional], Croatian, Czech, Danish, Dutch [Belgium, Netherlands], English, Spanish [Argentina, Colombia, Cuba, Mexico, Spain], Filipino, Finnish, French, German, Greek, Hebrew, Hindi, Hungarian, isiXhosa, isiZulu, Italian, Japanese, Korean, Lithuanian, Nepali, Persian, Polish, Portuguese [Brazil, Portugal], Romanian, Russian, Slovakian, Serbian, Swedish, Turkish, Urdu, Vietnamese). The translations were completed by a forward translator from the original English version, and then validated through both panel and back-translation processes by separate translators when possible.

### Data cleaning

Along with the original data file (COVIDiSTRESS global survey May 30 2020 (choice text).csv), we provide a cleaned data file (COVIDiSTRESS_May_30_cleaned_final.csv) where some cases were removed, and the issues regarding the coding of certain answers were corrected. R code used to clean the data is available online at the Open Science Framework (COVIDiSTRESS global survey^30^) and in supplementary information. The corrections made were:

- Filtered out cases without consent and younger than 18 years old.
- User Language – Bulgarian (BG): For responses between 2020-03-28 13:30:02 UTC and 2020-04-08 01:53:18 UTC, the order of the variable Country was mixed up for people who took the survey in Bulgarian language. Thus, the data was recoded.
- User Language – Afrikaans (AFR): For responses before 2020-04-07 06:48:00, the order of the variable Country was mixed up for people who took the survey in Afrikaans language. Thus, the data was recoded.
- User Language – Hebrew (HE): The variable Country was translated and arranged according to the Hebrew alphabetical order. Thus, the data was recoded.
- User Language – Bengali (BAN): Variables Scale_PSS10_UCLA_6 and Scale_PSS10_UCLA_7 were swapped during translation, so they were swapped back in the data cleaning procedure.
- Country: Removed dashes in front of the ‘- other’ responses in Country.
- Start Date: Cases before the official launch date 2020-03-30 were excluded as they were test answers. Soft launch answers from Denmark and Kosovo before the start date were retained.
- Marital Status: Except for the original English version of the survey, the order of the Dem_maritalstatus variable was mixed up in translations. The variable was recoded to correct this problem. There were some participants who had ‘5’ in Dem_maritalstatus. These responses were recoded as ‘Uninformative response’.
- Education level and mother’s education level: Removed dashes in front of the response options. There were some participants who had ‘1’ in Dem_edu. These responses were recoded as ‘Uninformative response’.
- Gender: The variable Dem_gender was inverted for languages SSP (Spanish - Spain) and SME (Spanish - Mexico) in the raw data file. Thus, in these responses, Male was recorded to Female and vice versa.
- AD_Check, AD_gain and AD_loss: Shorten the response; PSS-10, Corona_concerns, Compliance, BFF, SPS-10, Coping, Expl_media, Distress scale, Trust in the country’s measures: Responses were converted from choice text to numeric.
- Perceived Loneliness: The scale was initially coded as an extension of the PSS-10 battery. For clarity, the columns were renamed into Scale_PSS10_UCLA_11 through Scale_PSS10_UCLA_13 to Scale_Lon_1 through Scale_Lon_3.
- Created composite scores: PSS-10, SPS-10, SLON-3, BFF-15.
- Removed all new lines and “;” from participants’ additional text responses.
- From 15th May onwards, additional items (Q50-Q62) were included for a location-specific sub-study on war trauma in Bosnia/Herzegovina. These were not part of our pre-registration. These columns were cleaned (see below), but not included in the current report:

- Renamed new columns for clarity (Q50-Q62): born_92, experience_war, experience_war_TXT, war_injury, loss_during_war, time_spent_in_war, time_spent_in_war_TXT, Scale_UCLA_TRI_1:4 (4 items), PS_PTSD_1:5 (5 items)
- War-related questions: Removed numbers, periods, and extra spaces in the responses for the experience_war, war_injury, loss_during_war, time_spent_in_war (i.e. “2. Yes” got simplified to “Yes”)
- TRI_4: Responses were converted from choice text to numeric and composite score for the scale was calculated
- PS-PTSD: Responses were converted from choice text to numeric

Note that correcting the error-coded variables (Gender, User Language Bulgarian, Afrikaans and Hebrew, Marital Status) is necessary for correct interpretation of the data. None of the other actions described above (e.g., recoding text into numerical values) affect the data interpretation in any way. Apart from filtering out test data (data before the official launch on 2020-03-30) and participants who declared that they are younger than 18, all data was retained. When recoding, all groups present in the raw data file were also preserved. For more details, please see the data cleaning R markdown file. Thereafter, the text description is based on the cleaned data.

## Data Records

**Fig. 1:**
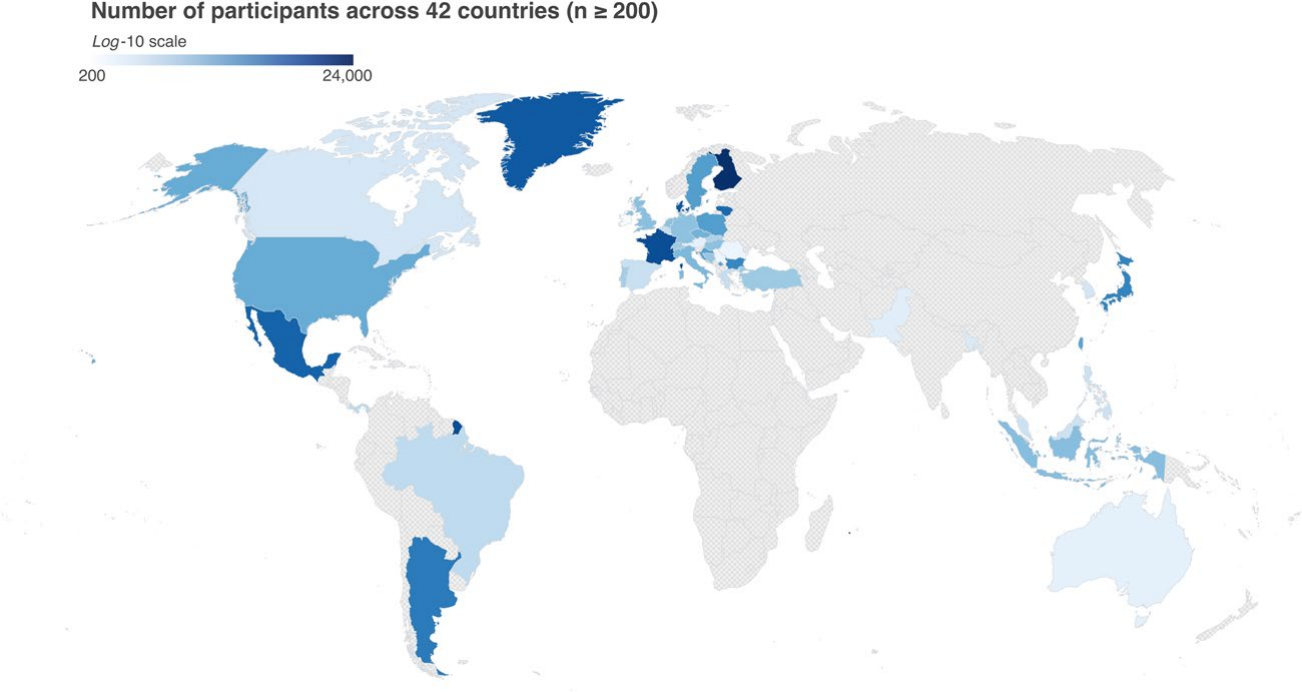
A world map visualizing the participants in each country. Only countries with n ≥ 200 are coloured.

Raw data and code for cleaning is available at 10.17605/OSF.IO/Z39US30. Figure 1 shows a heat map of the countries from which the data were collected, coloured according to the sample size (n ≥ 200). The main characteristics of the survey are presented in Tables 1 to 6. Information on the basics (Table 1), gender (Table 2), education (Table 3), marital status (Table 4), current risk of infection (Table 5), and current isolation status (Table 6) for countries with their sample size of more than 200 are presented, respectively.

**Table 2:** Proportion of each gender across countries with more than 200 participants.

Country	Prop_female	Prop_male	Prop_gender_other/not_say	Prop_gender_NA
Finland	0.813	0.167	0.018	0.002
France	0.510	0.472	0.016	0.002
Denmark	0.783	0.211	0.004	0.002
Mexico	0.720	0.270	0.006	0.005
Lithuania	0.751	0.242	0.006	0.001
Argentina	0.837	0.151	0.010	0.002
Japan	0.445	0.0.541	0.013	0.001
Bulgaria	0.807	0.172	0.019	0.002
Poland	0.867	0.125	0.008	0
Sweden	0.755	0.233	0.010	0.002
Croatia	0.783	0.212	0.003	0.002
Taiwan	0.700	0.273	0.026	0.001
Kosovo	0.629	0.356	0.011	0.004
United States	0.758	0.221	0.019	0.002
Czech Republic	0.782	0.212	0.006	0.001
Italy	0.761	0.229	0.007	0.003
Indonesia	0.671	0.307	0.016	0.006
United Kingdom	0.766	0.227	0.005	0.001
Germany	0.681	0.302	0.015	0.003
Hungary	0.707	0.287	0.003	0.003
Netherlands	0.738	0.251	0.008	0.0.003
Bosnia and Herzegovina	0.746	0.242	0.005	0.007
Turkey	0.746	0.239	0.013	0.002
Switzerland	0.608	0.382	0.003	0.007
Portugal	0.858	0.138	0.003	0.002
Slovakia	0.753	0.240	0.007	0
Panama	0.752	0.233	0.007	0.008
Brazil	0.735	0.254	0.004	0.007
Greece	0.760	0.232	0.005	0.003
Belgium	0.566	0.424	0.006	0.003
Spain	0.693	0.299	0.003	0.005
Philippines	0.665	0.318	0.018	0
Malaysia	0.741	0.247	0.009	0.004
Korea, South	0.466	0.522	0.008	0.004
Canada	0.668	0.302	0.026	0.004
Bangladesh	0.463	0.527	0.010	0
Pakistan	0.675	0.317	0.003	0.006
Australia	0.746	0.242	0.012	0
Austria	0.693	0.292	0.013	0.003
Romania	0.738	0.252	0.007	0.004
Serbia	0.673	0.316	0.008	0.004
Ireland	0.810	0.176	0.014	0

Note.

Prop_female = proportion of females.

Prop_male = proportion of males.

Prop_gender_other/not_say = proportion of participants of other genders or participants who did not want to declare their gender.

Prop_gender_NA = proportion of missing data for gender.

**Table 3:** Proportion of education level across countries with more than 200 participants.

Country	Prop_none	Prop_6years	Prop_9years	Prop_12years	Prop_some_college	Prop_college	Prop_PhD	Prop_edu_NA	Prop_uninf
Finland	0.023	0.070	0.032	0.137	0.205	0.484	0.041	0.008	0
France	0.009	0.012	0.008	0.082	0.212	0.577	0.098	0.003	0
Denmark	0.004	0.007	0.031	0.107	0.447	0.366	0.034	0.003	0.002
Mexico	0.004	0.001	0.006	0.053	0.093	0.540	0.300	0.003	0
Lithuania	0.001	0	0.003	0.030	0.144	0.769	0.052	0.003	0
Argentina	0.023	0.003	0.011	0.089	0.503	0.275	0.083	0.013	0
Japan	0.083	0.003	0.034	0.242	0.122	0.488	0.026	0.003	0
Bulgaria	0.003	0.000	0.002	0.104	0.228	0.599	0.047	0.015	0
Poland	0.004	0.000	0.008	0.151	0.232	0.547	0.051	0.006	0
Sweden	0.002	0.002	0.019	0.173	0.170	0.552	0.080	0.003	0
Croatia	0.003	0.002	0.006	0.163	0.164	0.608	0.051	0.004	0
Taiwan	0	0.000	0.003	0.046	0.034	0.619	0.297	0.001	0
Kosovo	0.004	0.000	0.003	0.160	0.564	0.242	0.019	0.007	0
United States	0.002	0.000	0.002	0.035	0.187	0.568	0.204	0.002	0
Czech Republic	0.004	0.001	0.004	0.150	0.276	0.515	0.046	0.004	0
Italy	0.019	0.003	0.020	0.201	0.237	0.437	0.070	0.013	0
Indonesia	0.001	0.001	0.003	0.086	0.110	0.760	0.040	0	0
United Kingdom	0	0.003	0.004	0.061	0.183	0.614	0.135	0	0
Germany	0.001	0.001	0.008	0.122	0.201	0.553	0.111	0.003	0
Hungary	0.001	0.002	0.021	0.339	0.307	0.287	0.039	0.006	0
Netherlands	0	0.002	0.001	0.016	0.141	0.770	0.061	0.008	0
Bosnia and Herzegovina	0.003	0.001	0.003	0.166	0.174	0.593	0.047	0.012	0
Turkey	0.003	0.003	0.004	0.047	0.059	0.554	0.328	0.003	0
Switzerland	0.005	0.003	0.030	0.129	0.171	0.566	0.090	0.006	0
Portugal	0.001	0.009	0.045	0.201	0.259	0.437	0.029	0.019	0
Slovakia	0.010	0.011	0.002	0.115	0.170	0.575	0.104	0.014	0
Panama	0.007	0.001	0.007	0.036	0.083	0.466	0.389	0.012	0
Brazil	0	0	0.004	0.031	0.342	0.506	0.114	0.003	0
Greece	0.005	0.005	0.008	0.083	0.128	0.600	0.154	0.019	0
Belgium	0.002	0.010	0.021	0.092	0.228	0.576	0.071	0.002	0
Spain	0.010	0.020	0.041	0.133	0.228	0.470	0.085	0.015	0
Philippines	0.002	0.005	0.007	0.065	0.521	0.367	0.030	0.004	0
Malaysia	0	0	0.002	0.011	0.093	0.596	0.296	0.002	0
Korea, South	0.002	0.002	0.004	0.033	0.043	0.686	0.228	0.002	0
Canada	0.006	0.006	0.004	0.045	0.230	0.566	0.143	0	0
Bangladesh	0.010	0	0	0.036	0.043	0.879	0.026	0.007	0
Pakistan	0	0	0.006	0.064	0.114	0.733	0.072	0.011	0
Australia	0.006	0	0.006	0.067	0.190	0.563	0.165	0.003	0
Austria	0.003	0	0.016	0.082	0.138	0.602	0.154	0.006	0
Romania	0.004	0	0.007	0.131	0.082	0.684	0.089	0.004	0
Serbia	0.004	0.004	0.008	0.113	0.387	0.421	0.064	0	0
Ireland	0	0.005	0.019	0.088	0.319	0.500	0.065	0.005	0

Note.

Prop_none = proportion of participants that have no education.

Prop_6years = proportion of participants that have up to 6 years of education.

Prop_9years = proportion of participants that have up to 9 years of education.

Prop_12years = proportion of participants that have up to 12 years of education.

Prop_some_college = proportion of participants that have finished some years of college or equivalent.

Prop_college = proportion of participants who have bachelor’s or master’s degrees.

Prop_PhD = proportion of participants who have PhD.

Prop_edu_NA = proportion of participants who have missing data for education variable.

Prop_uninf = proportion of participants with uninformative answers (answer coding errors).

**Table 4:** Proportion of marital status across countries with more than 200 participants.

Country	Prop_single	Prop_married/cohabiting	Prop_divorced/widowed	Prop_marital_other/not_say	Prop_marital_NA
Finland	0.205	0.632	0.106	0.051	0.006
France	0.495	0.418	0.045	0.036	0.006
Denmark	0.233	0.669	0.074	0.013	0.006
Mexico	0.505	0.397	0.076	0.020	0.003
Lithuania	0.208	0.655	0.089	0.042	0.006
Argentina	0.420	0.411	0.109	0.050	0.009
Japan	0.366	0.529	0.066	0.034	0.006
Bulgaria	0.265	0.547	0.128	0.044	0.017
Poland	0.223	0.711	0.025	0.037	0.005
Sweden	0.170	0.675	0.081	0.068	0.007
Croatia	0.352	0.511	0.050	0.069	0.017
Taiwan	0.657	0.272	0.015	0.052	0.005
Kosovo	0.576	0.355	0.013	0.043	0.012
United States	0.302	0.588	0.089	0.018	0.003
Czech Republic	0.396	0.505	0.076	0.019	0.005
Italy	0.301	0.502	0.102	0.091	0.004
Indonesia	0.479	0.475	0.021	0.018	0.007
United Kingdom	0.263	0.619	0.079	0.037	0.003
Germany	0.357	0.550	0.049	0.038	0.007
Hungary	0.179	0.632	0.156	0.026	0.006
Netherlands	0.235	0.652	0.068	0.038	0.007
Bosnia and Herzegovina	0.320	0.506	0.085	0.068	0.021
Turkey	0.515	0.399	0.050	0.028	0.008
Switzerland	0.394	0.485	0.088	0.027	0.006
Portugal	0.538	0.359	0.066	0.031	0.007
Slovakia	0.239	0.596	0.124	0.032	0.010
Panama	0.431	0.473	0.072	0.016	0.008
Brazil	0.529	0.363	0.089	0.016	0.003
Greece	0.512	0.167	0.033	0.280	0.008
Belgium	0.434	0.460	0.058	0.039	0.010
Spain	0.369	0.499	0.080	0.044	0.008
Philippines	0.732	0.191	0.026	0.039	0.012
Malaysia	0.531	0.407	0.034	0.018	0.011
Korea, South	0.439	0.501	0.025	0.025	0.010
Canada	0.311	0.568	0.087	0.028	0.006
Bangladesh	0.544	0.425	0.007	0.021	0.002
Pakistan	0.658	0.311	0.011	0.011	0.008
Australia	0.229	0.584	0.122	0.055	0.009
Austria	0.254	0.633	0.063	0.041	0.009
Romania	0.252	0.660	0.014	0.064	0.011
Serbia	0.365	0.519	0.068	0.049	0
Ireland	0.227	0.676	0.056	0.037	0.005

Note.

Prop_single = proportion of participants who are single.

Prop_married/cohabiting = proportion of participants who are married or cohabiting.

Prop_divorced/widowed = proportion of participants who are divorced or widowed.

Prop_marital_other/not_say = proportion of participants who live in some other form of community or don’t want to state their marital status.

Prop_marital_NA = proportion of missing data for the marital status variable.

**Table 5:** Proportion of current risk of infection across countries with more than 200 participants.

Country	Prop_yes	Prop_not_sure	Prop_no	Prop_NA
Finland	0.785	0.057	0.157	0.001
France	0.660	0.087	0.250	0.003
Denmark	0.615	0.069	0.303	0.013
Mexico	0.765	0.049	0.181	0.005
Lithuania	0.667	0.098	0.234	0.001
Argentina	0.782	0.049	0.166	0.003
Japan	0.346	0.101	0.553	0.001
Bulgaria	0.610	0.120	0.265	0.004
Poland	0.857	0.028	0.115	0.000
Sweden	0.757	0.043	0.198	0.002
Croatia	0.694	0.079	0.222	0.005
Taiwan	0.474	0.120	0.402	0.003
Kosovo	0.421	0.140	0.434	0.005
United States	0.750	0.040	0.207	0.003
Czech Republic	0.694	0.068	0.236	0.003
Italy	0.659	0.071	0.264	0.006
Indonesia	0.564	0.173	0.257	0.006
United Kingdom	0.625	0.070	0.303	0.002
Germany	0.649	0.082	0.265	0.003
Hungary	0.776	0.054	0.168	0.003
Netherlands	0.707	0.043	0.243	0.007
Bosnia and Herzegovina	0.620	0.088	0.283	0.009
Turkey	0.761	0.068	0.165	0.006
Switzerland	0.649	0.075	0.269	0.007
Portugal	0.790	0.056	0.152	0.002
Slovakia	0.626	0.085	0.287	0.002
Panama	0.700	0.043	0.237	0.020
Brazil	0.900	0.018	0.078	0.004
Greece	0.698	0.072	0.226	0.005
Belgium	0.635	0.098	0.264	0.003
Spain	0.691	0.068	0.231	0.010
Philippines	0.496	0.135	0.368	0
Malaysia	0.552	0.097	0.344	0.007
Korea, South	0.366	0.012	0.612	0.010
Canada	0.685	0.066	0.249	0
Bangladesh	0.399	0.264	0.337	0
Pakistan	0.386	0.150	0.458	0.006
Australia	0.697	0.049	0.248	0.006
Austria	0.608	0.088	0.295	0.009
Romania	0.681	0.106	0.206	0.007
Serbia	0.624	0.068	0.305	0.004
Ireland	0.750	0.051	0.199	0

Note.

Prop_yes = proportion of participants whose own or family members are at high risk,

Prop_not_sure = proportion of participants who are not sure,

Prop_no = proportion of participants whose own or family members are not at high risk.

Prop_NA = proportion of missing data for the risk variable.

**Table 6:** Proportion of current isolation status across countries with more than 200 participants.

Country	Prop_usual	Prop_minor	Prop_medical	Prop_isolated	Prop_NA
Finland	0.034	0.604	0.001	0.355	0.006
France	0.046	0.643	0.001	0.302	0.009
Denmark	0.020	0.658	0.001	0.318	0.004
Mexico	0.023	0.322	0.003	0.644	0.007
Lithuania	0.033	0.761	0.001	0.196	0.008
Argentina	0.024	0.320	0.001	0.625	0.029
Japan	0.474	0.513	0.001	0.011	0.002
Bulgaria	.031	0.399	0.001	0.537	0.031
Poland	0.012	0.450	0.001	0.524	0.013
Sweden	0.027	0.735	0	0.234	0.003
Croatia	.018	0.677	0.000	0.282	0.023
Taiwan	0.182	0.805	0.001	0.009	0.003
Kosovo	0.029	0.327	0.007	0.525	0.113
United States	0.016	0.380	0.001	0.597	0.005
Czech Republic	0.022	0.788	0	0.187	0.003
Italy	0.023	0.613	0.002	0.341	0.021
Indonesia	0.055	0.595	0.001	0.342	0.008
United Kingdom	0.020	0.400	0.001	0.572	0.007
Germany	0.030	0.586	0	0.366	0.017
Hungary	0.032	0.592	0.001	0.336	0.038
Netherlands	0.023	0.671	0	0.290	0.016
Bosnia and Herzegovina	0.041	0.580	0.003	0.355	0.021
Turkey	0.013	0.257	0.004	0.691	0.035
Switzerland	.031	0.642	0.001	0.318	0.008
Portugal	0.014	0.340	0.001	0.620	0.024
Slovakia	0.034	0.695	0	0.262	0.008
Panama	0.082	0.617	0.004	0.278	0.020
Brazil	0.008	0.283	0.003	0.699	0.007
Greece	0.012	0.343	0.005	0.625	0.016
Belgium	0.034	0.559	0.002	0.381	0.024
Spain	0.039	0.353	0.002	0.592	0.015
Philippines	0.121	0.761	0.002	0.111	0.005
Malaysia	0.093	0.670	0.002	0.229	0.005
Korea, South	0.230	0.743	0	0.018	0.008
Canada	0.015	0.396	0.004	0.579	0.006
Bangladesh	0.081	0.584	0.029	0.302	0.005
Pakistan	0.072	0.461	0.008	0.456	0.003
Australia	0.043	0.529	0	0.422	0.006
Austria	0.025	0.467	0	0.498	0.009
Romania	0.074	0.553	0	0.330	0.043
Serbia	0.030	0.519	0	0.440	0.011
Ireland	0.009	0.417	0	0.551	0.023

Note.

Prop_usual = proportion of participants whose life carries on as usual.

Prop_minor = proportion of participants whose life carries on with minor changes.

Prop_medical = proportion of participants who are isolated in medical facility or similar location.

Prop_isolated = proportion of participants who are isolated.

Prop_NA = proportion of missing data for the isolation variable.

### Data visualization interface

In addition to the raw data, a dedicated Web application was developed to provide a general overview of the COVIDiSTRESS dataset (https://covidistress.france-bioinformatique.fr/). The Web application allows easy and dynamic generation of illustrations like age pyramids, zoomable world maps, and bar plots summarizing the main variables of the survey for each selected country. Two tabs of visualizations are provided: the first contains basic demographic variables like age, gender, and educational level by country; the second tab displays world maps of levels of stress, trust in institutions and concerns for self, friends, family, country, and other countries. The application is based on an R shiny server (https://rstudio.com/products/shiny/shiny-server/), together with the plot.ly^31^ and ggplot2^32^ graphical libraries to generate dynamic plots. All the generated figures can be exported as PNG files.

## Technical Validation

As of 30th May, the participants in our data represented 176 different countries. However, there were instances in which we only had one participant per country (i.e. The Bahamas, Uganda, etc.). For computational purposes, we decided to examine the data quality for 42 countries that had over 200 participants.

**Fig. 2:**
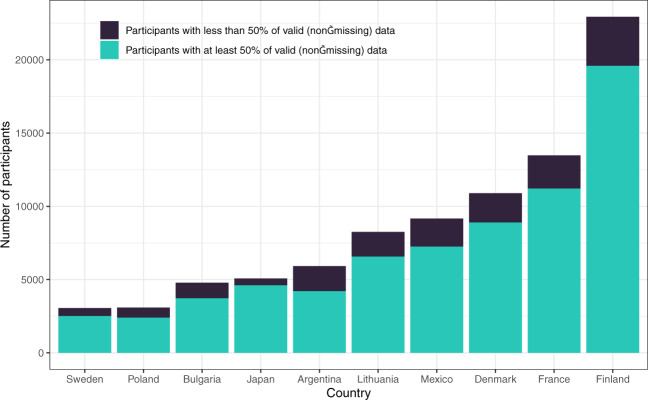
The number of participants and proportions of valid data across ten countries with the largest samples.

**Fig. 3:**
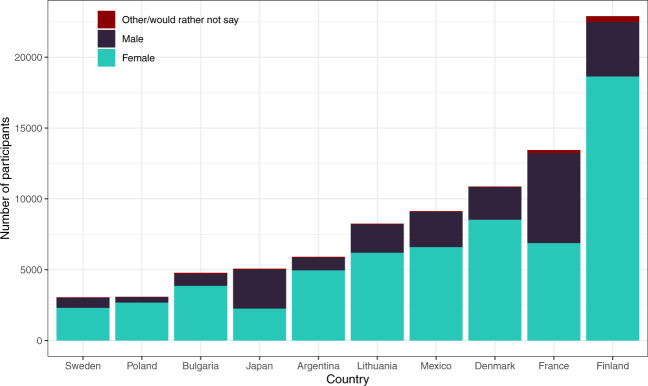
The distribution of gender across ten countries with the largest samples (missing data were excluded from this depiction due to very low proportions).

**Fig. 4:**
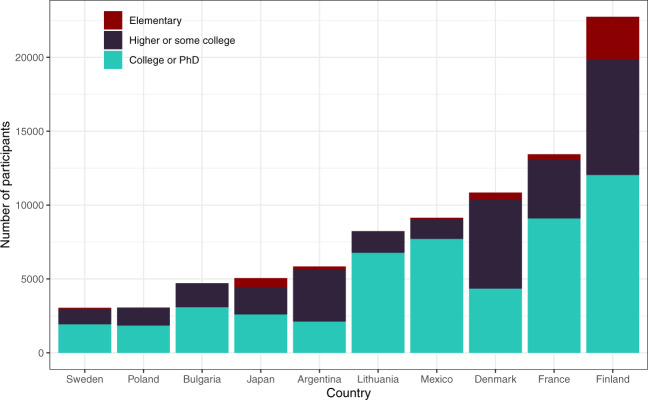
The distribution of education across ten countries with the largest samples (missing data were excluded from this depiction due to very low proportions).

Overall, 25 of these 42 countries had more than 1,000 participants. Among these, Finland, France, and Denmark are the three countries with the highest numbers of respondents (over 10,000). At least 62% of the participants provided answers to half of the questions in the survey, and at least 47% responded to 90% of the questions. We added one variable, “answered_all,” that indicates whether a participant answered all questions for users’ information. Of all 125,360 participants included in the cleaned dataset, 42.48% answered all questions. Figure 2 demonstrates the proportion of valid data across 10 countries with the highest number of participants (top 10 countries). The mean age of participants (M = 39.22, SD = 14.09) falls between young- to mid-adulthood, and in most countries, the number of female participants is disproportionately higher. Figure 3 illustrates the distribution of gender in the top 10 countries. Similarly, our sample seems to disproportionately represent people with some levels of higher education (i.e. some college or higher). Figure 4 shows participants’ levels of education in the top 10 countries. Additional details on the sample characteristics (including age, gender, education level, and marital status) can be found in Table 1 through Table 4. The dataset also includes answers to questions related to the respondent’s current likelihood of infection (e.g. risk of infection with COVID-19 in the family and the degree of isolation), as shown in Tables 5 and 6. Given our narrow timeline and the convenience sampling method, we acknowledge that our samples may not be representative of the populations of interest. However, we believe that the data can still be meaningfully used to understand the experiences of certain groups of people during this pandemic.

**Fig. 5:**
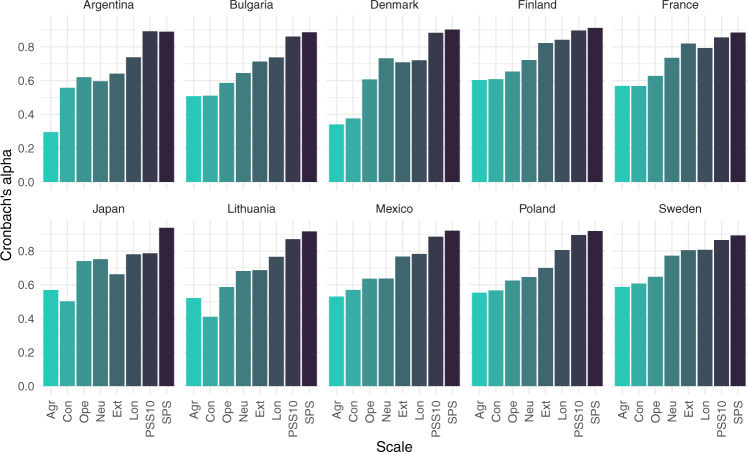
The Cronbach’s alpha reliability for each scale across ten countries with the largest samples.

Aside from some specific questions on COVID-19 (i.e. self-protective behaviours, trust in the government’s agencies, etc.), our data includes several scales that were previously validated within certain populations, including the Asian Disease Problem, PSS-10, SPS-10, BFF-15 (BFI-S), and the SLON-3. Figure 5 illustrates Cronbach’s alphas for these scales in the top 10 countries. In Table 7, we presented several descriptive statistics of each of the aforementioned continuous scales. Below, we described the preliminary statistics of the scales for all 42 countries.

**Table 7:** Descriptive statistics for continuous scales across all 42 countries with more than 200 participants.

Scale	pop_nonmis	Mean	SD	Min	Max	Alpha
PSS-10	0.898	2.632	734	1	5	0.873
SPS-10	735	4.904	851	1	6	0.920
SLON-3	0.913	2.566	0.994	1	5	771
BFI-S extraversion	0.859	3.950	1.118	1	6	751
BFI-S neuroticism	0.860	3.338	1.052	1	6	0.695
BFI-S openness	859	4.508	921	1	6	0.656
BFI-S agreeableness	0.860	4.433	825	1	6	0.535
BFI-S conscientiousness	0.859	4.356	883	1	6	591

Note.

Prop_nonmis = proportion of participants that responded to each scale.

Alpha = Cronbach’s alpha.

### Asian disease problem

The basic descriptive statistics of the Asian Disease Problem are summarized in Table 8. Specifically, among the 42 countries, at least 91% of the participants responded to this problem. They were randomly assigned to either of the gain or loss condition. Among those who responded, 50.27% were assigned to the gain condition, while 49.73% to the loss condition. Participants in the gain condition selected one of two options, Program A vs. B. Program A was selected by 66.20% of the participants in the gain condition, while 33.80% selected Program B. Those in the loss condition selected one of two options, Program C vs. D. Program C was selected by 36.54% of the participants in the loss condition, while 63.46% selected Program D.

**Table 8:** Descriptive statistics for the Asian Disease Problem across countries with more than 200 participants.

Country	N	Prop_nonmis	Prop_gain	Prop_program_A	Prop_program_B	Prop_loss	Prop_program_C	Prop_program_D
Argentina	5923	0.847	0.502	0.595	0.405	0.498	0.361	0.639
Australia	327	0.905	0.514	0.684	0.316	0.486	0.271	0.729
Austria	319	0.893	0.488	0.640	0.360	0.512	0.288	0.712
Bangladesh	421	0.805	0.507	0.616	0.384	0.493	0.269	0.731
Belgium	622	0.931	0.504	0.671	0.329	0.496	0.443	0.557
Bosnia and Herzegovina	1288	0.866	0.513	0.591	0.409	0.487	0.353	0.647
Brazil	731	0.923	0.508	0.624	0.376	0.492	0.319	.681
Bulgaria	4785	0.871	0.506	0.614	0.386	0.494	0.308	0.692
Canada	470	0.915	0.505	0.664	0.336	0.495	0.366	0.634
Croatia	2965	0.898	.497	0.623	0.377	0.503	0.330	0.670
Czech Republic	1995	0.904	0.492	0.538	0.462	0.508	0.353	0.647
Denmark	10891	0.909	0.501	0.680	0.320	0.499	0.372	0.628
Finland	22933	0.926	0.502	0.742	0.258	0.498	0.407	0.593
France	13475	0.932	0.508	0.710	0.290	0.492	0.438	0.562
Germany	1443	0.920	0.507	0.618	0.382	0.493	0.318	0.682
Greece	642	0.891	0.516	0.664	0.336	0.484	0.361	0.639
Hungary	1438	0.889	0.495	0.645	0.355	0.505	0.342	0.658
Indonesia	1569	0.887	0.504	0.513	0.487	0.496	0.333	0.667
Ireland	216	0.870	0.457	0.663	0.337	0.543	0.333	0.667
Italy	1749	0.842	0.505	0.586	0.414	0.495	0.291	0.709
Japan	5072	0.954	0.507	0.751	0.249	0.493	0.338	0.662
Korea, South	487	0.924	0.511	0.665	0.335	0.489	0.345	0.655
Kosovo	2707	0.803	0.497	0.633	0.367	0.503	0.361	0.639
Lithuania	8255	0.937	0.502	0.626	0.374	0.498	0.302	0.698
Malaysia	567	0.903	0.494	0.557	0.443	0.506	0.382	0.618
Mexico	9169	0.909	0.509	0.593	0.407	0.491	0.371	0.629
Netherlands	1433	0.909	0.474	0.661	0.339	0.526	0.428	0.572
Pakistan	360	0.836	0.505	0.592	0.408	0.495	0.362	0.638
Panama	759	0.810	0.504	0.616	0.384	0.496	0.407	0.593
Philippines	570	0.912	0.508	0.591	0.409	0.492	0.238	0.762
Poland	3088	0.935	0.500	0.600	0.0.400	0.500	0.236	0.764
Portugal	1067	0.906	0.499	0.671	0.329	0.501	0.287	0.713
Romania	282	0.840	0.519	0.569	0.431	0.481	0.298	0.702
Serbia	266	0.865	0.535	0.553	0.447	0.465	0.355	0.645
Slovakia	942	0.904	0.491	0.639	0.361	0.509	0.348	0.652
Spain	615	0.909	0.508	0.673	0.327	0.492	0.349	0.651
Sweden	3055	0.882	0.509	0.693	0.307	0.491	0.393	0.607
Switzerland	1188	0.912	0.505	0.676	0.0.324	0.495	0.437	0.563
Taiwan	2745	0.961	0.487	0.501	0.499	0.513	0.320	0.680
Turkey	1199	0.921	0.493	0.577	0.423	0.507	0.239	0.761
United Kingdom	1500	0.915	0.524	0.690	0.310	0.476	0.371	0.629
United States	2314	0.922	0.488	0.701	0.299	0.512	0.375	0.625

Note.

N = number of participants

Prop_nonmis = proportion of participants that responded to Asian Disease Problem.

Prop_gain = proportion of participants assigned to the gain condition among those responded to Asian Disease Problem.

Prop_program_A = proportion of participants who selected Program A among those assigned to the gain condition.

Prop_program_B = proportion of participants who selected Program B among those assigned to the gain condition.

Prop_loss = proportion of participants assigned to the loss condition among those responded to Asian Disease Problem.

Prop_program_C = proportion of participants who selected Program C among those assigned to the loss condition.

Prop_program_C = proportion of participants who selected Program D among those assigned to the loss condition.

### PSS-10

The basic descriptive statistics of the PSS-10 are summarized in Table 9. Specifically, among the 42 countries, at least 75% of the participants rated this scale. The composite scale score ranges from 1 to 5, with a mean value falling between 2.30 and 3.13. The internal consistency of the scale, as measured by Cronbach’s alpha, ranges from 0.66 to 0.90.

**Table 9:** Descriptive statistics and Cronbach’s alpha for the PSS across countries with more than 200 participants.

Country	N	Prop_nonmis	Mean	SD	Min	Max	Alpha
Argentina	5923	0.868	2.783	0.785	1.000	5	0.892
Australia	327	0.887	2.618	0.761	1.000	5	0.896
Austria	319	0.868	2.611	0.729	1.000	4.5	0.866
Bangladesh	421	0.808	2.830	0.592	1.000	4.3	0.794
Belgium	622	0.913	2.582	0.731	1.000	4.5	0.858
Bosnia and Herzegovina	1288	0.862	2.843	0.670	1.000	5	0.853
Brazil	731	0.896	3.059	0.730	1.100	5	0.882
Bulgaria	4785	0.859	2.848	0.719	1.000	5	0.861
Canada	470	0.894	2.715	0.723	1.000	5	0.880
Croatia	2965	0.890	2.875	0.661	1.000	5	0.860
Czech Republic	1995	0.882	2.694	0.707	1.000	4.9	0.878
Denmark	10891	0.910	2.423	0.717	1.000	5	0.883
Finland	22933	0.923	2.441	0.740	1.000	5	0.897
France	13475	0.905	2.564	0.742	1.000	5	0.856
Germany	1443	0.909	2.606	0.692	1.000	5	0.851
Greece	642	0.907	2.721	0.680	1.000	4.9	0.854
Hungary	1438	0.875	2.739	0.592	1.000	5	0.848
Indonesia	1569	0.857	2.749	0.591	1.000	5	0.837
Ireland	216	0.866	2.528	0.703	1.000	4.9	0.877
Italy	1749	0.893	2.539	0.687	1.000	5	0.861
Japan	5072	0.940	3.019	0.572	1.000	5	0.787
Korea, South	487	0.815	2.709	0.656	1.000	4.9	0.873
Kosovo	2707	0.809	2.861	0.541	1.000	5	0.666
Lithuania	8255	0.894	2.504	0.683	1.000	5	0.870
Malaysia	567	0.877	2.713	0.706	1.000	4.7	0.881
Mexico	9169	0.911	2.723	0.736	1.000	5	0.885
Netherlands	1433	0.909	2.298	0.677	1.000	4.6	0.885
Pakistan	360	0.747	2.883	0.718	1.000	5	0.816
Panama	759	0.851	2.430	0.632	1.000	4.7	0.852
Philippines	570	0.904	3.067	0.624	1.143	5	0.831
Poland	3088	0.892	2.993	0.729	1.000	5	0.894
Portugal	1067	0.882	2.886	0.726	1.100	5	0.884
Romania	282	0.869	2.668	0.651	1.000	4.6	0.881
Serbia	266	0.891	2.712	0.664	1.200	4.4	0.849
Slovakia	942	0.876	2.680	0.676	1.000	4.7	0.866
Spain	615	0.880	2.638	0.732	1.000	5	0.873
Sweden	3055	0.908	2.452	0.687	1.000	5	0.865
Switzerland	1188	0.918	2.378	0.650	1.000	4.5	0.831
Taiwan	2745	0.882	2.686	0.725	1.000	5	0.889
Turkey	1199	0.878	3.128	0.684	1.000	5	0.883
United Kingdom	1500	0.878	2.711	0.743	1.000	4.7	0.884
United States	2314	0.913	2.734	0.744	1.000	5	0.890

Note.

N = number of participants.

Prop_nonmissing = proportion of participants that have data on all items of the scale.

Mean = scale mean.

SD = scale standard deviation.

Min = minimal value of the average scale score.

Max = maximal value of the average scale score.

Alpha = Cronbach’s alpha.

### SPS-10

The basic descriptive statistics of the SPS-10 are summarized in Table 10. Specifically, among the 42 countries, at least half of the participants rated this scale. The composite scale score ranges from 1 to 6, with a mean value falling between 3.55 and 5.20. The internal consistency of the scale, as measured by Cronbach’s alpha, ranges from 0.88 to 0.94.

**Table 10:** Descriptive statistics and Cronbach’s alpha for the SPS across countries with more than 200 participants.

Country	N	Prop_nonmis	Mean	SD	Min	Max	Alpha
Argentina	5923	0.595	4.833	0.833	1.000	6	0.890
Australia	327	0.749	4.936	0.875	1.000	6	0.933
Austria	319	0.652	5.184	0.681	2.200	6	0.895
Bangladesh	421	0.558	4.806	0.770	2.100	6	0.901
Belgium	622	0.756	4.860	0.803	1.000	6	0.885
Bosnia and Herzegovina	1288	0.686	4.885	0.786	1.000	6	0.906
Brazil	731	0.688	5.167	0.710	2.375	6	0.904
Bulgaria	4785	0.685	4.808	0.790	1.000	6	0.886
Canada	470	0.770	4.868	0.818	1.400	6	0.910
Croatia	2965	0.737	5.059	0.709	1.500	6	0.893
Czech Republic	1995	0.727	4.925	0.758	1.400	6	0.904
Denmark	10891	0.752	5.203	0.693	1.000	6	0.902
Finland	22933	0.796	5.026	0.786	1.000	6	0.912
France	13475	0.770	4.881	0.805	1.000	6	0.884
Germany	1443	0.749	5.091	0.746	1.200	6	0.901
Greece	642	0.754	5.020	0.691	2.200	6	0.891
Hungary	1438	0.663	4.819	0.791	1.000	6	0.893
Indonesia	1569	0.611	4.590	0.741	1.000	6	0.892
Ireland	216	0.694	5.045	0.702	2.800	6	0.897
Italy	1749	0.735	4.891	0.736	1.000	6	0.891
Japan	5072	0.874	3.548	0.995	1.000	6	0.937
Korea, South	487	0.682	4.722	0.786	1.000	6	0.904
Kosovo	2707	0.498	4.881	0.717	1.700	6	0.878
Lithuania	8255	0.728	4.954	0.710	1.000	6	0.916
Malaysia	567	0.711	4.725	0.799	1.000	6	0.918
Mexico	9169	0.713	5.107	0.803	1.000	6	0.921
Netherlands	1433	0.752	5.029	0.690	1.000	6	0.909
Pakistan	360	0.525	4.750	0.822	1.100	6	0.908
Panama	759	0.631	5.187	0.726	1.100	6	0.914
Philippines	570	0.786	4.684	0.891	1.000	6	0.936
Poland	3088	0.690	5.000	0.743	1.000	6	0.918
Portugal	1067	0.627	5.109	0.682	1.900	6	0.893
Romania	282	0.624	4.890	0.766	2.200	6	0.909
Serbia	266	0.729	5.016	0.709	2.800	6	0.890
Slovakia	942	0.669	4.862	0.790	1.000	6	0.914
Spain	615	0.676	4.970	0.832	1.200	6	0.904
Sweden	3055	0.765	5.119	0.701	1.300	6	0.892
Switzerland	1188	0.765	5.120	0.717	1.000	6	0.901
Taiwan	2745	0.754	4.373	0.856	1.000	6	0.910
Turkey	1199	0.689	4.935	0.805	1.000	6	0.909
United Kingdom	1500	0.706	4.991	0.750	1.700	6	0.906
United States	2314	0.779	5.109	0.758	1.000	6	0.920

Note.

N = number of participants.

Prop_nonmis = proportion of participants that have data on all items of the scale.

Mean = scale mean.

SD = scale standard deviation.

Min = minimal value of the average scale score.

Max = maximal value of the average scale score.

Alpha = Cronbach’s alpha.

### SLON-3

The basic descriptive statistics of the SLON-3 are summarized in Table 11. Specifically, among the 42 countries, at least 77% of the participants rated this scale. The composite scale score ranges from 1 to 5, with a mean value falling between 1.89 and 3.05. The internal consistency of the scale, as measured by Cronbach’s alpha, ranges from 0.54 to 0.84.

**Table 11:** Descriptive statistics and Cronbach’s alpha for the SLON across countries with more than 200 participants.

Country	N	Prop_nonmis	Mean	SD	Min	Max	Alpha
Argentina	5923	0.889	2.626	1.036	1.000	5	0.738
Australia	327	0.899	2.701	0.998	1.000	5	0.771
Austria	319	0.893	2.658	0.987	1.000	5	0.765
Bangladesh	421	0.824	2.790	0.856	1.000	5	0.576
Belgium	622	0.923	2.575	1.017	1.000	5	0.811
Bosnia and Herzegovina	1288	0.880	2.905	0.935	1.000	5	0.740
Brazil	731	0.912	2.755	0.913	1.000	5	0.714
Bulgaria	4785	0.884	2.743	1.020	1.000	5	0.737
Canada	470	0.904	2.726	0.959	1.000	5	0.765
Croatia	2965	0.902	2.901	0.894	1.000	5	0.737
Czech Republic	1995	0.893	2.952	0.971	1.000	5	0.761
Denmark	10891	0.922	2.308	0.890	1.000	5	0.720
Finland	22933	0.935	2.647	1.026	1.000	5	0.842
France	13475	0.923	2.420	1.027	1.000	5	0.793
Germany	1443	0.921	2.700	0.997	1.000	5	0.774
Greece	642	0.919	2.543	0.957	1.000	5	0.735
Hungary	1438	0.893	2.806	0.874	1.000	5	0.721
Indonesia	1569	0.872	2.352	0.952	1.000	5	0.799
Ireland	216	0.884	2.611	0.967	1.000	5	0.724
Italy	1749	0.916	2.757	0.973	1.000	5	0.776
Japan	5072	0.951	2.441	0.891	1.000	5	0.780
Korea, South	487	0.817	2.421	0.881	1.000	5	0.712
Kosovo	2707	0.839	2.324	0.884	1.000	5	0.618
Lithuania	8255	0.909	2.571	0.954	1.000	5	0.766
Malaysia	567	0.877	2.462	0.986	1.000	5	0.828
Mexico	9169	0.926	2.494	1.010	1.000	5	0.782
Netherlands	1433	0.911	2.491	0.887	1.000	5	0.786
Pakistan	360	0.769	2.712	1.052	1.000	5	0.699
Panama	759	0.881	2.220	0.837	1.000	5	0.675
Philippines	570	0.918	2.780	0.905	1.000	5	0.719
Poland	3088	0.905	3.052	1.047	1.000	5	0.806
Portugal	1067	0.898	2.592	0.939	1.000	5	0.721
Romania	282	0.879	2.868	0.899	1.000	5	0.724
Serbia	266	0.917	2.825	0.932	1.000	5	0.696
Slovakia	942	0.883	2.963	0.935	1.000	5	0.747
Spain	615	0.901	2.530	1.014	1.000	5	0.771
Sweden	3055	0.918	2.580	0.990	1.000	5	0.807
Switzerland	1188	0.929	2.468	0.936	1.000	5	0.764
Taiwan	2745	0.890	1.887	0.852	1.000	5	0.790
Turkey	1199	0.888	2.781	0.788	1.000	5	0.536
United Kingdom	1500	0.891	2.696	1.001	1.000	5	0.772
United States	2314	0.922	2.672	1.005	1.000	5	0.778

Note.

N = number of participants.

Prop_nonmissing = proportion of participants that have data on all items of the scale.

Mean = scale mean.

SD = scale standard deviation.

Min = minimal value of the average scale score.

Max = maximal value of the average scale score.

Alpha = Cronbach’s alpha.

### BFF-15

This term was used for this project. This is more commonly known as the Big Five Inventory-SOEP (BFI-S).

#### Extraversion

The basic descriptive statistics of this subscale are summarized in Table 12. Specifically, among the 42 countries, at least 71% of participants rated this scale. The composite subscale score ranges from 1 to 6, with a mean value falling between 3.12 to 4.50. The internal consistency of the scale, as measured by Cronbach’s alpha, ranges from 0.51 to 0.86.

**Table 12:** Descriptive statistics and Cronbach’s alpha for the BFI-S extraversion scale across countries with more than 200 participants.

Country	N	Prop_nonmis	Mean	SD	Min	Max	Alpha
Argentina	5923	0.810	3.953	1.002	1.000	6	0.641
Australia	327	0.847	3.786	1.184	1.000	6	0.816
Austria	319	0.796	4.315	1.088	1.000	6	0.813
Bangladesh	421	0.732	4.130	1.061	1.000	6	0.746
Belgium	622	0.867	3.847	1.198	1.000	6	0.792
Bosnia and Herzegovina	1288	0.844	4.444	0.988	1.000	6	0.755
Brazil	731	0.832	4.195	1.062	1.000	6	0.766
Bulgaria	4785	0.838	4.500	0.967	1.000	6	0.713
Canada	470	0.855	3.672	1.143	1.000	6	0.808
Croatia	2965	0.857	4.351	1.009	1.000	6	0.775
Czech Republic	1995	0.834	3.852	1.098	1.000	6	0.820
Denmark	10891	0.877	4.190	1.005	1.000	6	0.709
Finland	22933	0.891	4.148	1.132	1.000	6	0.823
France	13475	0.871	3.796	1.196	1.000	6	0.820
Germany	1443	0.865	4.009	1.109	1.000	6	0.782
Greece	642	0.861	4.353	1.012	1.000	6	0.765
Hungary	1438	0.800	4.226	1.035	1.000	6	0.728
Indonesia	1569	0.790	3.843	.965	1.000	6	0.694
Ireland	216	0.843	3.986	1.081	1.333	6	0.749
Italy	1749	0.870	4.005	1.063	1.000	6	0.765
Japan	5072	0.924	3.117	0.905	1.000	6	0.662
Korea, South	487	0.791	3.513	0.882	1.000	6	0.506
Kosovo	2707	0.752	4.156	0.877	1.000	6	0.526
Lithuania	8255	0.855	3.473	1.009	1.000	6	0.686
Malaysia	567	0.802	3.482	1.071	1.000	6	0.768
Mexico	9169	0.849	3.710	1.145	1.000	6	0.767
Netherlands	1433	0.862	4.082	1.029	1.000	6	0.774
Pakistan	360	0.708	3.916	1.079	1.333	6	0.670
Panama	759	0.810	3.807	1.022	1.000	6	0.647
Philippines	570	0.877	3.668	1.084	1.000	6	0.733
Poland	3088	0.836	3.926	0.999	1.000	6	0.700
Portugal	1067	0.813	4.266	1.057	1.000	6	0.794
Romania	282	0.812	4.199	1.048	1.333	6	0.788
Serbia	266	0.868	4.072	0.941	1.333	6	0.632
Slovakia	942	0.804	4.025	1.000	1.000	6	0.751
Spain	615	0.836	4.139	1.083	1.000	6	0.738
Sweden	3055	0.881	4.205	1.027	1.000	6	0.805
Switzerland	1188	0.882	4.202	1.053	1.000	6	0.794
Taiwan	2745	0.863	3.536	1.148	1.000	6	0.861
Turkey	1199	0.808	4.502	1.003	1.000	6	0.757
United Kingdom	1500	0.839	3.870	1.100	1.000	6	0.768
United States	2314	0.872	3.810	1.203	1.000	6	0.827

Note.

N = number of participants.

Prop_nonmissing = proportion of participants that have data on all items of the scale.

Mean = scale mean.

SD = scale standard deviation.

Min = minimal value of the average scale score.

Max = maximal value of the average scale score.

Alpha = Cronbach’s alpha.

#### Neuroticism

The basic descriptive statistics of this subscale are summarized in Table 13. Specifically, among the 42 countries, at least 70% of the participants rated this scale. The composite subscale score ranges from 1 to 6, with a mean value falling between 2.91 and 3.80. The internal consistency of the scale, as measured by Cronbach’s alpha, ranges from 0.44 to 0.77.

**Table 13:** Descriptive statistics and Cronbach’s alpha for the BFI-S neuroticism scale across countries with more than 200 participants.

Country	N	Prop_nonmis	Mean	SD	Min	Max	Alpha
Argentina	5923	0.819	3.763	0.968	1.000	6	0.596
Australia	327	0.844	3.292	1.060	1.000	6	0.709
Austria	319	0.796	3.054	0.973	1.000	5.66667	0.702
Bangladesh	421	0.739	3.197	0.972	1.000	6	0.610
Belgium	622	0.868	3.277	1.018	1.000	6	0.670
Bosnia and Herzegovina	1288	0.843	3.136	0.978	1.000	6	0.646
Brazil	731	0.833	3.602	1.110	1.000	6	0.713
Bulgaria	4785	0.842	3.048	1.002	1.000	6	0.645
Canada	470	0.857	3.439	1.033	1.000	6	0.726
Croatia	2965	0.858	3.204	0.994	1.000	6	0.702
Czech Republic	1995	0.834	3.597	0.994	1.000	6	0.736
Denmark	10891	0.877	2.962	1.087	1.000	6	0.732
Finland	22933	0.892	3.092	1.040	1.000	6	0.722
France	13475	0.872	3.535	1.109	1.000	6	0.735
Germany	1443	0.866	3.167	1.036	1.000	6	0.733
Greece	642	0.860	3.565	0.979	1.000	6	0.590
Hungary	1438	0.809	3.308	0.983	1.000	6	0.664
Indonesia	1569	0.791	3.625	0.767	1.000	6	0.440
Ireland	216	0.838	3.353	1.001	1.000	6	0.715
Italy	1749	0.867	3.358	0.985	1.000	6	0.613
Japan	5072	0.928	3.793	0.982	1.000	6	0.752
Korea, South	487	0.784	3.335	0.916	1.000	6	0.571
Kosovo	2707	0.758	3.387	1.002	1.000	6	0.630
Lithuania	8255	0.856	3.419	0.949	1.000	6	0.681
Malaysia	567	0.804	3.666	0.828	1.667	6	0.506
Mexico	9169	0.852	3.571	0.978	1.000	6	0.637
Netherlands	1433	0.862	2.967	1.026	1.000	6	0.749
Pakistan	360	0.703	3.802	0.920	1.000	6	0.444
Panama	759	0.814	3.362	0.867	1.000	6	0.482
Philippines	570	0.879	3.725	0.883	1.000	6	0.508
Poland	3088	0.838	3.497	0.956	1.000	6	0.646
Portugal	1067	0.812	3.763	1.143	1.000	6	0.763
Romania	282	0.812	3.270	0.987	1.000	6	0.669
Serbia	266	0.872	3.330	0.874	1.333	5.66667	0.525
Slovakia	942	0.809	3.359	0.991	1.000	6	0.762
Spain	615	0.833	3.440	1.058	1.000	6	0.680
Sweden	3055	0.883	2.905	1.026	1.000	6	0.772
Switzerland	1188	0.875	2.937	1.013	1.000	6	0.711
Taiwan	2745	0.863	3.802	0.919	1.000	6	0.690
Turkey	1199	0.810	3.422	1.025	1.000	6	0.674
United Kingdom	1500	0.840	3.361	1.026	1.000	6	0.698
United States	2314	0.869	3.420	1.028	1.000	6	0.693

Note.

N = number of participants.

Prop_nonmissing = proportion of participants that have data on all items of the scale.

Mean = scale mean.

SD = scale standard deviation.

Min = minimal value of the average scale score.

Max = maximal value of the average scale score.

Alpha = Cronbach’s alpha.

#### Openness

The basic descriptive statistics of this subscale are summarized in Table 14. Specifically, among the 42 countries, at least 71% of the participants rated this scale. The composite subscale score ranges from 1 to 6, with a mean value falling between 3.36 and 4.97. The internal consistency of the scale, as measured by Cronbach’s alpha, ranges from 0.46 to 0.74.

**Table 14:** Descriptive statistics and Cronbach’s alpha for the BFI-S openness scale across countries with more than 200 participants.

Country	N	Prop_nonmis	Mean	SD	Min	Max	Alpha
Argentina	5923	0.814	4.762	0.861	1.000	6	0.620
Australia	327	0.847	4.528	0.862	1.667	6	0.608
Austria	319	0.799	4.711	0.832	1.333	6	0.597
Bangladesh	421	0.734	4.580	0.704	2.000	6	0.504
Belgium	622	0.870	4.525	0.907	1.000	6	0.611
Bosnia and Herzegovina	1288	0.839	4.668	0.812	1.333	6	0.604
Brazil	731	0.836	4.586	0.898	1.667	6	0.620
Bulgaria	4785	0.843	4.706	0.816	1.000	6	0.586
Canada	470	0.853	4.635	0.881	1.667	6	0.629
Croatia	2965	0.858	4.649	0.820	1.333	6	0.628
Czech Republic	1995	0.832	4.417	0.821	1.000	6	0.616
Denmark	10891	0.875	4.352	0.983	1.000	6	0.607
Finland	22933	0.891	4.664	0.879	1.000	6	0.653
France	13475	0.870	4.431	0.945	1.000	6	0.628
Germany	1443	0.866	4.631	0.864	1.000	6	0.653
Greece	642	0.868	4.556	0.814	1.667	6	0.525
Hungary	1438	0.806	4.113	0.857	1.000	6	0.474
Indonesia	1569	0.788	4.576	0.706	1.000	6	0.618
Ireland	216	0.833	4.321	0.902	1.000	6	0.606
Italy	1749	0.860	4.514	0.872	1.000	6	0.584
Japan	5072	0.927	3.364	0.938	1.000	6	0.740
Korea, South	487	0.786	4.403	0.900	1.667	6	0.693
Kosovo	2707	0.752	4.618	0.762	1.000	6	0.472
Lithuania	8255	0.856	4.436	0.829	1.000	6	0.586
Malaysia	567	0.804	4.365	0.765	2.000	6	0.500
Mexico	9169	0.849	4.886	0.769	1.000	6	0.636
Netherlands	1433	0.864	4.391	0.879	1.000	6	0.579
Pakistan	360	0.706	4.595	0.789	2.000	6	0.456
Panama	759	0.808	4.968	0.740	1.000	6	0.643
Philippines	570	0.881	4.396	0.925	1.000	6	0.646
Poland	3088	0.838	4.436	0.857	1.333	6	0.625
Portugal	1067	0.812	4.401	0.885	1.000	6	0.587
Romania	282	0.812	4.538	0.821	1.000	6	0.593
Serbia	266	0.872	4.587	0.806	2.333	6	0.649
Slovakia	942	0.805	4.622	0.775	1.000	6	0.649
Spain	615	0.828	4.693	0.862	1.667	6	0.689
Sweden	3055	0.882	4.449	0.908	1.000	6	0.647
Switzerland	1188	0.877	4.517	0.852	1.333	6	0.592
Taiwan	2745	0.863	4.200	0.847	1.000	6	0.660
Turkey	1199	0.808	4.721	0.814	1.333	6	0.706
United Kingdom	1500	0.842	4.557	0.851	1.500	6	0.601
United States	2314	0.870	4.652	0.840	2.000	6	0.611

Note.

N = number of participants.

Prop_nonmissing = proportion of participants that have data on all items of the scale.

Mean = scale mean.

SD = scale standard deviation.

Min = minimal value of the average scale score.

Max = maximal value of the average scale score.

Alpha = Cronbach’s alpha.

#### Agreeableness

The basic descriptive statistics of this subscale are summarized in Table 15. Specifically, among the 42 countries, at least 71% of participants rated this scale. The composite subscale score ranges from 1 to 6, with a mean value falling between 3.62 and 4.85. The internal consistency of the scale, as measured by Cronbach’s alpha, ranges from 0.30 to 0.67.

**Table 15:** Descriptive statistics and Cronbach’s alpha for the BFI-S agreeableness scale across countries with more than 200 participants.

Country	N	Prop_nonmis	Mean	SD	Min	Max	Alpha
Argentina	5923	0.817	4.523	0.807	1.000	6	0.295
Australia	327	0.847	4.484	0.814	2.333	6	0.576
Austria	319	0.796	4.414	0.766	2.333	6	0.497
Bangladesh	421	0.736	4.351	0.766	1.000	6	0.424
Belgium	622	0.867	4.451	0.841	1.000	6	0.581
Bosnia and Herzegovina	1288	0.845	4.563	0.778	1.000	6	0.569
Brazil	731	0.834	4.346	0.812	1.667	6	0.501
Bulgaria	4785	0.843	4.382	0.849	1.333	6	0.508
Canada	470	0.851	4.539	0.781	1.333	6	0.546
Croatia	2965	0.859	4.482	0.759	1.333	6	0.550
Czech Republic	1995	0.832	4.049	0.816	1.333	6	0.526
Denmark	10891	0.875	4.549	0.750	1.333	6	0.340
Finland	22933	0.891	4.517	0.781	1.000	6	0.604
France	13475	0.872	4.421	0.872	1.000	6	0.569
Germany	1443	0.868	4.351	0.803	1.000	6	0.531
Greece	642	0.866	4.663	0.726	2.000	6	0.466
Hungary	1438	0.806	4.301	0.832	1.000	6	0.562
Indonesia	1569	0.790	4.322	0.751	2.000	6	0.397
Ireland	216	0.847	4.527	0.764	2.000	6	0.413
Italy	1749	0.866	4.451	0.810	1.000	6	0.518
Japan	5072	0.927	3.619	0.757	1.000	6	0.568
Korea, South	487	0.786	4.351	0.807	1.667	6	0.668
Kosovo	2707	0.757	4.854	0.742	1.000	6	0.536
Lithuania	8255	0.857	4.245	0.778	1.000	6	0.522
Malaysia	567	0.810	4.372	0.783	1.333	6	0.539
Mexico	9169	0.852	4.604	0.833	1.000	6	0.530
Netherlands	1433	0.863	4.672	0.732	1.000	6	0.538
Pakistan	360	0.711	4.391	0.751	2.000	6	0.450
Panama	759	0.809	4.740	0.834	1.333	6	0.471
Philippines	570	0.879	4.481	0.780	1.333	6	0.444
Poland	3088	0.838	4.292	0.755	1.000	6	0.553
Portugal	1067	0.813	4.491	0.780	2.000	6	0.496
Romania	282	0.809	4.529	0.733	1.667	6	0.481
Serbia	266	0.876	4.483	0.799	2.333	6	0.531
Slovakia	942	0.807	4.583	0.779	1.000	6	0.583
Spain	615	0.837	4.607	0.810	1.667	6	0.461
Sweden	3055	0.881	4.707	0.727	1.667	6	0.587
Switzerland	1188	0.883	4.391	0.786	1.667	6	0.531
Taiwan	2745	0.863	4.154	0.833	1.000	6	0.646
Turkey	1199	0.808	4.405	0.849	1.333	6	0.544
United Kingdom	1500	0.841	4.485	0.799	1.667	6	0.547
United States	2314	0.869	4.571	0.791	1.667	6	0.564

Note.

N = number of participants.

Prop_nonmissing = proportion of participants that have data on all items of the scale.

Mean = scale mean.

SD = scale standard deviation.

Min = minimal value of the average scale score.

Max = maximal value of the average scale score.

Alpha = Cronbach’s alpha.

#### Conscientiousness

The basic descriptive statistics of this subscale are summarized in Table 16. Specifically, among the 42 countries, at least 70% of participants rated this scale. The composite subscale score ranges from 1 to 6, with a mean value falling between 3.54 and 5.01. The internal consistency of the scale, as measured by Cronbach’s alpha, ranges 0.34 to 0.67.

**Table 16:** Descriptive statistics and Cronbach’s alpha for the BFI-S conscientiousness scale across countries with more than 200 participants.

Country	N	Prop_nonmis	Mean	SD	Min	Max	Alpha
Argentina	5923	0.817	4.766	0.825	1.000	6	0.558
Australia	327	0.841	4.350	0.800	2.000	6	0.509
Austria	319	0.799	4.556	0.841	1.667	6	0.669
Bangladesh	421	0.736	4.116	0.834	1.500	6	0.536
Belgium	622	0.865	4.129	0.901	1.667	6	0.542
Bosnia and Herzegovina	1288	0.839	4.714	0.782	2.000	6	0.603
Brazil	731	0.832	4.089	0.811	1.000	6	0.352
Bulgaria	4785	0.841	4.884	0.732	1.667	6	0.511
Canada	470	0.851	4.363	0.879	1.333	6	0.641
Croatia	2965	0.858	4.585	0.813	1.333	6	0.635
Czech Republic	1995	0.833	3.814	0.800	1.000	6	0.510
Denmark	10891	0.869	4.576	0.756	1.000	6	0.376
Finland	22933	0.891	4.375	0.844	1.000	6	0.608
France	13475	0.872	4.054	0.929	1.000	6	0.568
Germany	1443	0.868	4.329	0.854	1.000	6	0.588
Greece	642	0.861	4.267	0.779	1.667	6	0.411
Hungary	1438	0.806	4.406	0.867	1.000	6	0.628
Indonesia	1569	0.790	3.993	0.792	1.667	6	0.575
Ireland	216	0.843	4.418	0.869	2.000	6	0.650
Italy	1749	0.864	4.318	0.840	1.333	6	0.487
Japan	5072	0.928	3.536	0.777	1.000	6	0.502
Korea, South	487	0.789	4.123	0.863	1.333	6	0.667
Kosovo	2707	0.752	4.760	0.728	1.000	6	0.404
Lithuania	8255	0.856	4.087	0.757	1.000	6	0.410
Malaysia	567	0.804	4.226	0.804	1.000	6	0.546
Mexico	9169	0.849	4.796	0.804	1.000	6	0.570
Netherlands	1433	0.864	4.561	0.741	1.000	6	0.483
Pakistan	360	0.697	4.218	0.825	2.333	6	0.475
Panama	759	0.812	5.009	0.724	2.333	6	0.545
Philippines	570	0.877	4.036	0.789	1.000	6	0.460
Poland	3088	0.837	4.217	0.815	1.000	6	0.566
Portugal	1067	0.807	4.160	0.760	2.000	6	0.339
Romania	282	0.801	4.394	0.788	1.667	6	0.457
Serbia	266	0.868	4.324	0.823	1.667	6	0.624
Slovakia	942	0.811	4.345	0.810	1.000	6	0.581
Spain	615	0.834	4.578	0.841	1.667	6	0.545
Sweden	3055	0.878	4.530	0.785	1.667	6	0.607
Switzerland	1188	0.881	4.534	0.813	1.333	6	0.583
Taiwan	2745	0.862	3.622	0.832	1.000	6	0.542
Turkey	1199	0.809	4.533	0.820	1.333	6	0.518
United Kingdom	1500	0.835	4.395	0.824	1.000	6	0.576
United States	2314	0.868	4.515	0.800	1.667	6	0.548

Note.

N = number of participants.

Prop_nonmissing = proportion of participants that have data on all items of the scale.

Mean = scale mean.

SD = scale standard deviation.

Min = minimal value of the average scale score.

Max = maximal value of the average scale score.

Alpha = Cronbach’s alpha.

## Usage Notes

We recommend that any interested researchers use the raw or the cleaned version of the latest extracted data (available at 10.17605/OSF.IO/Z39US). The data was imported and cleaned using the *R* software for statistical analysis^33^ and packages *tidyverse*^34^*, multicon*^35^*, qualtRics*^36^*, pacman*^37^*, and psych*^38^. Before using the dataset, the steps in the Data cleaning section should be followed to ensure that the dataset is ready for analysis. The data cleaning procedure should involve excluding irrelevant cases, correcting some errors in value-coding, and renaming improperly named variables. In addition, the cleaning procedure should encompass recoding choice values to number, creating composite scores, and the estimation of the Cronbach alpha reliabilities for the measured scales (PSS-10, BFF-15, SPS-10, and SLON-3). However, for analysis in individual countries, we recommend checking for tau-equivalence before using Cronbach’s alpha for reliability estimation. If tau-equivalence is not achieved, Omega coefficient is more appropriate as a reliability indicator^39,40^. Before analysing the data, it should be noted that the answers in variables measuring distress (‘Expl_Distress_*no*’) are recoded to numeric values 1, 2, 3, 4, 5, and 6, measuring the degree of agreement, and 99, which means that the item does not apply to one’s current situation. Additionally, answers in the variable ‘Trust_countrymeasure’ are recoded on a scale from 0 to 10, where 0 and 10 suggest inappropriate measures (too little or too much) and values around 5 suggest appropriate measures.

To merge the present dataset with a pre-existing cross-cultural dataset by country and date, the variables ‘Country’ and ‘RecordedDate’ should be used.

Finally, the samples in the present dataset are not representative of the populations from which they are drawn (in each country). Thus, users who wish to address this issue may weigh the data by referring to demographic information for each country and apply the appropriate weights for the variables and countries of interest (e.g., age: http://data.un.org/Data.aspx?d=POP&f=tableCode%3A22; gender: https://ourworldindata.org/gender-ratio; education: https://ourworldindata.org/global-education; marital status: https://ourworldindata.org/marriages-and-divorces).

## Supplementary information

Figure S1.

## Data Availability

Raw data and R-code for cleaning are available at 10.17605/OSF.IO/Z39US
