# Supplementary material for: COVIDiSTRESS Global Survey dataset on psychological and behavioural consequences of the COVID-19 outbreak
Source: Sci Data. 2021 Jan 4;8:3. doi: 10.1038/s41597-020-00784-9 (PMC7782539; doi:10.1038/s41597-020-00784-9)
Supplement: Supplementary file 1 — Figure S1. [file 41597_2020_784_MOESM1_ESM.pdf]

	Affiliation, Country	Conceptualization	Data curation	Formal analysis	Funding acqusition	Investigation	Methodology	Project administration	Resources	Software	Supervision	Validation	Visualization	Writing (original draft)	Writing (review & editing)	Funding Information
Yuki Yamada	Kyushu University, Faculty of Arts and Science, Japan				■	■		■					■	■		JSPS KAKENHI Grants JP17H00875, JP18K12015, JP20H04581
Dominik-Borna Ćepulić	Catholic University of Croatia, Department of Psychology		■			■							■	■		
Tao Coll-Martín	Mind, Brain, and Behavior Research Center (CIMCYC), University of Granada, Granada, Spain					■	■						■	■		
Stephane Debove	Independent researcher, France					■		■					■	■		
Gautreau Guillaume	Université Paris-Saclay, France												■	■		
Hyemin Han	University of Alabama, Educational Psychology Program, USA				■	■		■						■		
Jesper Rasmussen	Aarhus University, Department of Political Science, Denmark		■			■								■		
Thao P. Tran	Colorado State University, Psychology, USA		■			■							■			
Giovanni A. Travaglino	University of Kent, UK					■		■					■	■		
Andreas Lieberoth	Aarhus University, Danish School of Educaction (DPU) and Interacting Minds Center (IMC), Denmark	■	■		■	■	■	■	■	■	■			■		
**Consortium authors**																
Angélique M. Blackburn	Texas A&M International University, Department of Psychology & Communications, USA					■										
Loïs Boullu	Independant, France						■						■			
Mila Bujić	Tampere University, Faculty of Information Technology and Communication Sciences, Finland					■	■									
Grace Byrne	Developmental Psychology, Vrije Universiteit Amsterdam, the Netherlands		■			■										
Marjolein C.J. Caniëls	Open University, Faculty of Management sciences					■	■									
Ivan Flis	Catholic University of Croatia, Department of Psychology					■		■								
Marta Kowal	University of Wrocłąw, Institute of Psychology, Poland		■			■										
Nikolay R. Rachev	Sofia University St. Kliment Ohridski, Bulgaria, Department of General, Experimental, Developmental, and Health Psychology					■	■									
Vicenta Reynoso-Alcántara	University of Veracruz; National Autonomous University of Mexico					■	■									
Oulmann Zerhouni	Université Paris Nanterre					■		■								
Oli Ahmed	Department of Psychology, University of Chittagong, Bangladesh					■										
Rizwana Amin	Bahria University Islamabad, Dept of Professional Psychology, Pakistan					■										
Sibele Aquino	Pontifical Catholic University of Rio de Janeiro, Brazil					■										
João Carlos Areias	University of Porto, Faculty of Psychology and Educational Sciences, Portugal					■										
John Jamir Benzon R. Aruta	De La Salle University, Manila, Philippines					■										
Dastan Bamwesigye	Mendel University in Brno					■										
Jozef Bavolar	Pavol Jozef Safarik University, Faculty of Arts, Department of Psychology, Slovakia					■										
Andrew R. Bender	Michigan State University, Department of Epidemiology and Biostatistics, East Lansing, USA					■										
Pratik Bhandari	Department of Psychology, and Department of Language Science and Technology, Saarland University, Germany					■										
Tuba Bircan	Vrije UNiversiteit Brussel, Interface Demography					■										
Huseyin Cakal	School of Psychology, Keele University					■										
Tereza Capelos	University of Birmingham, UK					■										
Jiří Čeněk	Mendel University in Brno				■	■										Czech Science Foundation GC19-09265J
Brendan Ch'ng	Department of Educational Psychology and Counselling, Faculty of Education, University of Malaya					■										
Fang-Yu Chen	Michigan State University					■										
Stavroula Chrona	King's College London, School of Politics and Economics, Department of European and International Studies (EIS)					■										
Carlos C. Contreras-Ibáñez	Departamento de Sociología. Universidad Autónoma Metropolitana, Iztapalapa. Mexico					■										
Pablo Sebastián Correa	Instituto de Investigaciones Psicológicas (IIPsi), Universidad Nacional de Córdoba (UNC), Consejo Nacional de Investigaciones Científicas y Técnicas (CONICET), Argentina					■										
Irene Cristofori	Department of Biology, University Claude Bernard Lyon 1/ Institute of Cognitive Sciences Marc Jeannerod CNRS UMR5229						■							■		
Wilson Cyrus-Lai	INSEAD					■										
Guillermo Delgado-Garcia	Instituto Nacional de Neurologia y Neurocirugia, Mexico				■	■										Consejo Nacional de Ciencia y Tecnologia (Conacyt), Full National Scholarship - MSc degree (CVU: 613905)
Eliane Deschrijver	Ghent University, Department of Experimental Psychology, Belgium; University of New South Wales (UNSW), School of Psychology, Australia				■	■										Research Foundation Flanders (FWO) postdoctoral fellowship
Carlos Díaz	Independent researcher. Denmark					■										
İlknur Dilekler	TOBB University of Economics and Technology, Department of Psychology, Turkey					■										
Vilius Dranseika	Faculty of Social Sciences, Arts and Humanities, Kaunas University of Technology, Lithuania					■										
Dmitrii Dubrov	National Research University Higher School of Economics, Russian Federation				■	■										The HSE University Basic Research Program
Kristina Eichel	Department of Psychiatry and Human Behavior, Warren Alpert Medical School, Brown University, USA					■										
Eda Ermagan-Caglar	University of Northampton, Department of Psychology					■										
Rebekah Gelpí	University of Toronto, Department of Psychology					■										
Rubén Flores González	University of Veracruz					■										
Amanda Griffin	Univeristy of Oregon					■										
Moh Abdul Hakim	Department of Psychology, Universitas Sebelas Maret					■										
Krzysztof Hanusz	Institute of Psychology Polish Academy of Sciences					■										
Yuen Wan Ho	Northeastern University, Psychology Department, USA					■										
Dayana Hristova	Faculty of Psychology, University of Vienna					■										
Barbora Hubena	Independent researcher, Czech Republic					■										
Keiko Ihaya	Kyushu University, Admission Center, Japan				■	■										JSPS KAKENHI Grant JP20K14222
Gozde Ikizer	TOBB University of Economics and Technology, Department of Psychology, Turkey					■										
Md. Nurul Islam	University of Chittagong, Psychology, Bangladesh					■										
Alma Jeftic	Peace Research Institute, International Christian University, Tokyo					■										
Shruti Jha	Somerville School (Lott Carey Baptist Mission in India), Greater NOIDA, India					■										
Fernanda Pérez-Gay Juárez	McGill University, Canada					■										
Pavol Kacmar	Pavol Jozef Safarik University, Faculty of Arts, Department of Psychology, Slovakia					■										
Kalina Kalinova	Sofia University St. Kliment Ohridski, Bulgaria, Department of General, Experimental, Developmental, and Health Psychology					■										
Phillip S. Kavanagh	Discipline of Psychology, University of Canberra					■										
Mehmet Kosa	Tilburg University, Department of Cognitive Science and Artificial Intelligence, Netherlands					■										
Karolina Koszałkowska	University of Lodz, Institute of Psychology, Poland					■										
Raisa Kumaga	School of Health and Social Care,University of Essex, United Kingdom					■										
David Lacko	Masaryk university, Faculty of Arts, Department of Psychology					■										
Yookyung Lee	The University of Texas at Austin, Educational Psychology, USA					■										
Antonio G. Lentoor	Sefako Makgatho Health Sciences University, Department of Clinical Psychology, School of Medicine					■										
Gabriel A. De Leon	Texas A&M International University					■										
Shiang-Yi Lin	the Education University of Hong Kong, Centre for Child and Family Sciences, Hong Kong					■										
Samuel Lins	University of Porto, Faculty of Psychology and Educational Sciences, Portugal					■										
Claudio Rafael Castro López	University of Veracruz					■										
Agnieszka E. Lys	University of Warsaw, Faculty of Psychology					■										
Samkelisiwe Mahlungulu	Sefako Makgatho Health Sciences University, Department of Clinical Psychology, School of Medicine					■										
Tsvetelina Makaveeva	Sofia University St. Kliment Ohridski, Bulgaria, Department of General, Experimental, Developmental, and Health Psychology					■										
Salomé Mamede	University of Porto, Faculty of Psychology and Educational Sciences, Portugal					■										
Silvia Mari	University of Milano-Bicocca					■										
Tiago A. Marot	Pontifical Catholic University of Rio de Janeiro, Brazil					■										
Liz Martinez	University of California, Merced					■										
Dar Meshi	Michigan State University, Department of Advertising and Public Relations, USA					■										
Débora Jeanette Mola	Instituto de Investigaciones Psicológicas (IIPsi), Universidad Nacional de Córdoba (UNC), Consejo Nacional de Investigaciones Científicas y Técnicas (CONICET), Argentina					■										
Sara Morales-Izquierdo	University of Warwick, Department of Psychology					■										
Arian Musliu	Department of Psychology, Ludwig Maximilian University, Munich, Germany					■										
Priyanka A. Naidu	Griffith University, School of Applied Psychology, Australia					■										
Arooj Najmussaqib	Department of Professional Psychology, Bahria University Islamabad, Pakistan					■										
Jean C. Natividade	Pontifical Catholic University of Rio de Janeiro, Brazil					■										
Steve Nebel	Psychology of learning with digital media, Department of Media Research, Germany					■										
Jana Nezkusilova	Pavol Jozef Safarik University, Slovakia					■										
Irina Nikolova	Open University, Faculty of Management sciences					■										
Manuel Ninaus	Leibniz-Institut für Wissensmedien, Tübingen, Germany					■										
Valdas Noreika	Department of Psychology, University of Cambridge, United Kingdom					■										
María Victoria Ortiz	Instituto de Investigaciones Psicológicas (IIPsi), Universidad Nacional de Córdoba (UNC), Consejo Nacional de Investigaciones Científicas y Técnicas (CONICET), Argentina					■										
Daphna Hausman Ozery	California State University, Northridge, Department of Educational Psychology & Conseling					■										
Daniel Pankowski	University of Economics and Human Sciences in Warsaw, Faculty of Psychology					■										
Tiziana Pennato	School of Compared Psychotherapy, Florence, Italy					■										
Martin Pírko	Institute of Lifelong Learning at Mendel University in Brno					■										
Lotte Pummerer	Leibniz-Institut für Wissensmedien, Tübingen, Germany					■										
Cecilia Reyna	Instituto de Investigaciones Psicológicas (IIPsi), Universidad Nacional de Córdoba (UNC), Consejo Nacional de Investigaciones Científicas y Técnicas (CONICET), Argentina					■										
Eugenia Romano	King's College London, Psychological Medicine, Institute of Psychiatry, Psychology and Neurosciences					■										
Hafize Sahin	Independent researcher, UK					■										
Aybegum Memisoglu Sanli	Middle East Technical University, Department of Psychology					■										
Gülden Sayılan	Ankara Yıldırım Beyazıt University Department of Psychology, Turkey					■										
Alessia Scarpaci	Independent Researcher. Psychology and Cognitive Neuroscience. Italy/United Kingdom					■										
Cristina Sechi	Department of Pedagogy, Psychology, Philosophy					■										
Maor Shani	Hebrew University of Jerusalem, Israel					■										
Aya Shata	University of Miami, School of Communication, USA					■										
Pilleriin Sikka	University of Turku, Department of Psychology and Speech-Language Pathology, Finland; University of Skövde, Department of Cognitive Neuroscience and Philosophy, Sweden					■										
Nidhi Sinha	Indian Institute of Technology, Hyderabad, India					■										
Sabrina Stöckli	Department Consumer Behavior, University of Bern					■										
Anna Studzinska	University of Economics and Human Sciences in Warsaw, Faculty of Psychology					■										
Emilija Sungailaite	Independent, United Kindgom					■										
Zea Szebeni	University of Helsinki, Swedish School of Social Sciences					■										
Benjamin Tag	University of Melbourne					■										
Mihaela Taranu	Aarhus University, Insitute for Culture and Society, Interacting Minds centre					■										
Franco Tisocco	Instituto de Investigaciones en Psicología, Facultad de Psicología, Universidad de Buenos Aires					■										
Jarno Tuominen	University of Turku, Department of Psychology and Speech-Language Pathology, Finland					■										
Fidan Turk	University of Sheffield, Department of Psychology, UK					■										
Muhammad Kamal Uddin	University of Dhaka, Department of Psychology, Bangladesh					■										
Ena Uzelac	Faculty of Humanities and Social Sciences in Zagreb, Department of Psychology, Croatia					■										
Sara Vestergren	University of Salford, School of Health & Society, UK					■										
Roosevelt Vilar	Faculdades Integradas de Patos, Department of Psychology, Brazil					■										
Austin Horng-En Wang	University of Nevada, Las Vegas					■										
J. Noël West	University of Sheffield, Department of Philosophy					■										
Charles K.S. Wu	Department of Political Science, Purdue University					■										
Teodora Yaneva	Sofia University St. Kliment Ohridski, Bulgaria, Department of General, Experimental, Developmental, and Health Psychology					■										
Yao-Yuan Yeh	University of St. Thomas, Houston, Center for International Studies, USA					■										
